# Machine learning of big data in gaining insight into successful treatment of hypertension

**DOI:** 10.1002/prp2.396

**Published:** 2018-04-24

**Authors:** Gideon Koren, Galia Nordon, Kira Radinsky, Varda Shalev

**Affiliations:** ^1^ Maccabi-Kahn Institute of Research and Innovation and Tel Aviv University Tel Aviv Israel; ^2^ Technion-Israel Institute of Technology Haifa Israel

**Keywords:** beta blockers, big data, hypertension, machine learning, protein pump inhibitors, statins

## Abstract

Despite effective medications, rates of uncontrolled hypertension remain high. Treatment protocols are largely based on randomized trials and meta‐analyses of these studies. The objective of this study was to test the utility of machine learning of big data in gaining insight into the treatment of hypertension. We applied machine learning techniques such as decision trees and neural networks, to identify determinants that contribute to the success of hypertension drug treatment on a large set of patients. We also identified concomitant drugs not considered to have antihypertensive activity, which may contribute to lowering blood pressure (BP) control. Higher initial BP predicts lower success rates. Among the medication options and their combinations, treatment with beta blockers appears to be more commonly effective, which is not reflected in contemporary guidelines. Among numerous concomitant drugs taken by hypertensive patients, proton pump inhibitors (PPIs), and HMG CO‐A reductase inhibitors (statins) significantly improved the success rate of hypertension. In conclusions, machine learning of big data is a novel method to identify effective antihypertensive therapy and for repurposing medications already on the market for new indications. Our results related to beta blockers, stemming from machine learning of a large and diverse set of big data, in contrast to the much narrower criteria for randomized clinic trials (RCTs), should be corroborated and affirmed by other methods, as they hold potential promise for an old class of drugs which may be presently underutilized. These previously unrecognized effects of PPIs and statins have been very recently identified as effective in lowering BP in preliminary clinical observations, lending credibility to our big data results.

**Abbreviations**
ADMA: asymetric dimethylarginine
AUC: area under the receiver-operator curve
BP: blood pressure
DHD: Coronary Heart Disease
CVD: cardiovascular disease
PPI: proton pump inhibitor
RAS: renin-angiotensin system
RC4Ts: randomized clinic trials

## 1 INTRODUCTION

Hypertension is a very common medical condition, affecting roughly 20% of the world population.^1^ It is a leading cause of mortality and morbidity including stroke, heart failure, coronary artery disease, and chronic kidney disease. In addition to lifestyle modifications, it is typically treated with 1 or more classes of medications, which include thiazide‐diuretics, calcium channel blockers, angiotensin converting enzyme inhibitors, and angiotensin receptor blockers. Recommendations regarding the use of beta blockers are not consensual, with some guidelines recommending their use, other do not, and some only if other medications have failed to control blood pressure (BP).^2^ Expert groups in all parts of the world are continuously reviewing the evidence accumulating on success of hypertension therapy. While there is no consensus on the best initial treatment, it is widely recognized that most patients need medications from more than 1 class, but recommendations in treatment guidelines are heterogenous.

The existing recommendations are based on the available evidence, stemming mostly from RCTs and systematic reviews of RCTs. However, no RCTs have identified an optimal dosing or drug combination strategy.^2^

Most traditional algorithms in medicine are sets of rules based on existing knowledge in a specific topic (in our case hypertension). In contrast, machine learning algorithms are a relatively new area of research in computer sciences and statistics, aiming to identify novel and valid patterns in data. Machine learning encompasses different modeling tools, which utilize computers to uncover “hidden insights” through learning from trends in large sets of data.^3^

The objective of this study was to use this new approach to identify effective treatment choices for hypertension based on big data analysis of a large cohort of hypertensive patients. In parallel, it was aimed to identify concomitant drugs not taken for hypertension which may contribute to success in lowering BP.

## 2 MATERIALS AND METHODS

From the electronic medical charts of Maccabi Health Services, the second largest health service organization in Israel insuring more than 2 million members,^4^ we identified patients receiving their first ever drug treatment for hypertension after a diagnosis had been made. Medications utilized were identified from the electronically recorded purchases by the patient. For these patients, initial systolic and diastolic values were calculated using the mean of at least measurement over the 200 days before treatment. Patients lacking at least 2 measurements in this time frame were excluded. Weight, age, BMI, and smoking status were extracted from the electronic medical charts, calculating their mean, median, maximum, minimum, and standard deviation. The success criterion was defined as achieving BP lower than 140/90 in at least 1 measurement within 90 days of treatment initiation. Measurements were performed by primary care practitioners. No standardization of measurements among physicians was aimed.

### 2.1 Machine learning methodology

“Classification” is a task in machine learning in which the data can be divided into separate categories or classes. The algorithm is attempting to predict the correct class for each data item in the repository. In our case, there were 2 classes: “treatment success” (ie, achieving BP lower than 140/90 within 90 days of treatment initiation), and “treatment failure” (any other case).

We used 2 types of machine learning algorithms for this task: Decision trees^5^, ^6^ and fully connected Neural networks.^7^, ^8^ The analysis was done using Python, and statistical and machine learning functions and infrastructure from the following packages and libraries: Scipy, Sklearn, and Keras.

In the training phase of the machine learning model, the computer is presented with a training data set. For each example in this dataset the correct classification is also given. The model strives to set its internal variables in a way that minimizes the difference between its prediction and the correct classification for each example in the test set. In the case of Decision Tree algorithms, the internal variables of the model represent a tree structure in which a decision is made in each branch according to the data features. Ensemble methods such as random forest and xgboost create numerous variations in trees (a forest) and combine them into a single model. Neural network are built as networks of cells (neurons) where each cell preforms a simple mathematic operation, the weights given to each cell's output are adjusted in order to achieve the best prediction.

The performance of the model was tested using cross validation aiming to reduce overfitting, whereby 90% of the derivation data are used as a learning subset to construct a model, and examine its performance on the remaining 10%. This process was repeated 10 times by dividing the derivation set into new and different learning and testing subsets.

We calculated for each drug or their combinations the success rate and the area under the receiver‐operator curve (AUC), where the *x* axis marks the false positive rate (1 − specificity) and the *y* axis shows true positive rates (sensitivity). The “positive” set contained patients who received the drug treatment and met the criterion of “BP lower 140/90 within 90 days of treatment initiation”; and the “negative” set contained patients who received the drug treatment while NOT meeting the criterion. The closer the AUC is to 1.0, the better is the overall performance of the mode.^9^

In an attempt to eliminate as much patient variability among drug choices, we used propensity score matching to examine whether a specific drug treatment/combination achieved independently higher success rates.^10^ We used the following patient characteristics for the matching: hypertension drug treatment, initial BP, weight, age, BMI, and smoking status. Re‐sampling was allowed in the matching process (ie, the same patient could be matched to several patients from the original group). The basic idea behind matching is to try and match 1 group of observations with another group of observations in such a way that the items in the groups are as similar as possible in all aspects except for the tested variable. In our case, given a group of patients that are treated with drug x, we aimed to match every patient with a patient that is identical to him/her in age, weight, BMI, etc., except for the fact that the matched patient was not treated with drug x.

#### 2.1.1 Effects of concomitant drugs on hypertension

In addition to antihypertensive medications, our dataset contained records of all other purchases of prescribed pharmaceuticals given by the health care providers to hypertensive patients.

Patients from the untreated group were matched to patients from the treated group based on the propensity score.

We performed an exhaustive search over all treatment groups, excluding those that were bought by <200 patients, identifying 73 such groups. For each treatment group, we compared hypertension treatment success rates of the group of patients treated with that specific treatment and a matched group of patients that were not treated with that specific treatment. Based on the entire data base, logistic regression was used for predicting the probability of treatment success with the matched drug and this constituted the propensity score. For each patient in the treated group we matched a patient untreated with that specific treatment with the closest propensity score.

Pearson's chi‐squared test was used to determine whether the success rates differed among groups. To accommodate for multi hypothesis testing, the *P*‐values were corrected according to the Bonferoni correction. We present the 5 smallest chi square *P*‐values including the corrected *P*‐value (ie, original *P*‐value times number of hypotheses tested).

We used the following patient characteristics for the matching: hypertension drug treatment, initial BP, weight, age, BMI, and smoking status. Treatment groups were excluded according to rate of re‐sampling and Kelmogorov‐Smirnof (KS) goodness of fit tests for all features.^11^ We chose a re‐sampling rate of 20%, with *P* < .0001 for a single feature as our limit for group's exclusion. That is, if the KS test for 1 of the features we matched had *P* > .001, we considered it to be an ill fit and discarded the treatment group. However, given that other matching parameters were correct, such an ill‐fitted feature may be interpreted as another factor (in addition to the treatment group) for hypertension treatment success.

## 3 RESULTS

Based on our exclusion criteria, the resulting dataset contained 30 705 patients, whose characteristics are presented in Table 1. Most patients (17 234) were initially treated with 1 class of hypertensive drugs, 9176 were initially treated using 2 drug types, 3425 and 867 patient were treated with 3 and 4 drugs, respectively (Table 2). ACE inhibitors and ARB were the most common treatment, used by 73% of the patients. Beta blockers were prescribed to 47% of the patients making them the second most common treatment. These rates held either in overall prescriptions as well as when analyzing drug combinations (Table 3).

**Table 1:** Characteristics of patients who were successfully treated for hypertension within 90 days from diagnosis, as compared to unsuccessful cases

Parameter	Mean in successful treatment	Mean in unsuccessful treatment
Initial systolic BP	142.74	158.76
Initial diastolic BP	85.74	92.46
Systolic‐diastolic	57	66.3
Age	55.39	55.8
BMI	26.72	26.46
Weight	74.29	74.56
Smoking	2.34	2.21
Sex	1.44	1.51

BP, blood pressure.

**Table 2:** Distribution of patients according to the numbers treated with 1 drug type or combinations of 2, 3, or 4 drug types

No. of drugs	Total	Success rate (%)	AUC	ACE	Beta	Calcium	Diuretics
1 drug type	17 234	44	0.71	10 903	2853	2157	1321
2 drug types	9176	41	0.71	7325	4261	3830	2743
3 drug types	3425	40	0.71	3204	2599	2462	2015
4 drug types	867	38	0.72	867	867	867	867

AUC, area under the receiver‐operator curve.

**Table 3:** Numbers of patients treated with each antihypertensive drug, either alone or in combinations

Drug type	No. of patients	Success rate (%)	AUC
ACE	22 498	38.7	0.71
Beta	10 580	41.9	0.72
Calcium	9316	31.7	0.73
Diuretics	6941	36.4	0.71

AUC, area under the receiver‐operator curve.

Beta blockers had the highest success rate among the different drug groups either by themselves, in 2 drug combinations (Table 4), or in 3 drug combinations (Table 5).

**Table 4:** Success rates in 2 drug combinations

Drug type	Success rate (%)	AUC (avg. of 10)
ACE inhib. + another	40	0.70
Beta block. + another	46	0.71
Calcium ch. block. + another	32	0.79
Thiazide‐diuretics + another	39	0.71

AUC, area under the receiver‐operator curve.

**Table 5:** Success rates in 3 drug combinations

Drug type	Success rate (%)	AUC (avg. of 10)
ACE inhib. + others	39	0.71
Beta block. + others	43	0.68
Calcium ch. block. + others	32	0.72
Thiazide‐diuretics + others	40	0.68

AUC, area under the receiver‐operator curve.

We used 3 variations in decision tree classifiers for predicting treatment success: Decision tree, random forest, and xgboost. In all cases the maximal tree depth was set to 5 with a minimum of 100 samples per leaf. These classifiers achieved an average AUC of 0.7 (Table 6). The important predictors in all variations were as follows: initial systolic value, the difference between initial systolic and diastolic value and the initial diastolic value. The lower these observed values were, the greater the likelihood that treatment would prove successful. Additional important predicting features were as follows: weight, age, and BMI but these were less prominent.

**Table 6:** Decision tree classifiers for predicting treatment success

Classifier alg.	AUC (avg. of 10)
Decision tree	0.7
Random forest	0.68
XGBoost9	0.73

AUC, area under the receiver‐operator curve.

Table 7 describes the results of applying a different classification algorithm—Neural networks. We used a fully connected neural network with 1 hidden layer of size 146 (input size of 139) and a relu activation function. For the neural network we defined a different variation in the classification task: first treatment with treatment group drug (for each of the 4 groups). The performance for this algorithm was better‐up to 0.82 AUC, but the most predictive features remained the initial BP values. Removing these features decreased the network's performance to AUC of around 0.5.

**Table 7:** Neural network scores for different variations in classification tasks^a^

Classification task (neural network)	AUC (avg. of 10)
Any drug	0.8
ACE inhibitors	0.79
Beta blockers	0.8
Calcium channel blockers	0.82
Diuretics	0.82

AUC, area under the receiver‐operator curve.

a Classification task is “success” or “failure” in controlling blood pressure as defined in the methodology.

### 3.1 Effects of concomitant drugs on hypertension treatment success

As seen in Table 8, 2 groups of medications, proton pump inhibitors (PPI) and HMG CO‐A reductase Inhibitors (statins) were found to have highly significant improved success rates for hypertension treatment after Bonferoni correction for multiple comparisons.

**Table 8:** Success in causing an anti hypertensive effect by concomitant drugs not aimed for hypertension. Proton pump inhibitors and statins achieved the highest significance levels

Treatment group	Chi‐squared *P*‐value	Corrected *P*‐value
Proton pump inhibitors	<.3 × 10^−6^	<.3 × 10^−6^
HMG CO‐A reductase inhibitors	<.3 × 10^−7^	<7.2 × 10^−5^
Platelet aggregation inhibit	<1.6 × 10^−3^	<7 × 10^−1^
Antimycotic + steroid	<1.7 × 10^−2^	<.24
Corticosteroids, inhaler	<2.7 × 10^−2^	<.2

To account for multiple comparisons, a significant anti hypertensive effect was set on corrected *P* values of *P* < .001.

## 4 DISCUSSION

Similar to traditional regression models, in machine learning there are generally outcomes, covariates, and a statistical function linking the 2. Different from traditional statistics, machine learning considers large numbers of predictors by combining them in nonlinear and highly interactive computational methods. In the model construction phase of the forest algorithm, for example, the model automatically generates decision trees which aim at identifying success rates of treatment. The model's performance is tested by using 90% of the data for construction, and the remaining 10% for examination of its performance. This process is repeated 10 times by dividing the derivation set into new and different learning and testing subsets. The model created through these steps could then be applied on a new and previously unused data.^3^, ^9^

In our case, machine learning algorithms allowed us to identify novel and valid patterns in hypertension treatment data which cannot be figured out through traditional experimental or observational approaches. To date the published experimental data on hypertension have typically compared the effectiveness of drug A to drug B, but never combinations of 3 or 4 medications, The use of machine learning, encompassing different modeling tools, allowed us to uncover hidden insights and trends in the data using large amounts of “real world” data.

Our analysis reveals that the majority of patients needed combinations of drugs, with ACE inhibitors and angiotensin receptor blockers prevail both as the most commonly used single drug, as well as in combination with other antihypertensive medications. To date, all guidelines worldwide recommend ACEI, ARB, CCB, and diuretics, acknowledging that, because the main purpose is lowering BP per se, the drug groups should not be prioritize or differentiate among them.^2^ In contrast, beta blockers are recommended only by some expert bodies and national guidelines (European Society of Hypertension and Cardiology, France and China). Other guidelines, including NICE and ASH/ISH recommend beta blockers as an additional drug, when the other options have not been sufficiently effective.^2^ The lesser enthusiasm toward beta blockers stems from studies claiming it to be less effective than other classes in preventing stroke or other cardiovascular events, and causing a long list of adverse effects such as fatigue, sexual dysfunction, reduced exercise tolerance, and increased incidence of new onset diabetes.^2^ However, the latest (2017) Cochrane systematic review compared beta blockers as a single drug to other single drug therapies for hypertension, shedding light on the potential role of this class of drugs.^12^

Thirteen RCTs compared beta blockers to either placebo, diuretics, CCB, or renin‐angiotensin system (RAS) inhibitors. There were no differences in all‐cause mortality among beta blockers and placebo, diuretics, or RAS inhibitors, but it was higher for beta blockers compared to CCBs. Total cardiovascular disease (CVD) was lower for beta blockers compared to placebo, a reflection of the decrease in CVD. The effect of beta blockers on CVD was inferior to that of CCBs. In addition, there was an increase in stroke with beta blockers compared to CCBs and RAS inhibitors. However, there was little or no difference in between beta blockers and diuretics, CCBs, or RAS inhibitors. Patients taking beta blockers were more likely to discontinue treatment due to adverse events than patients taking RAS, but there was little or no difference when compared with placebo, diuretics, or CCBs.^12^

The common denominator of all these studies is that they compared beta blockers to other single drugs. In contrast, our study examined the effectiveness of combinations of antihypertensive drugs in successful treatment of hypertension. It shows that when a beta blocker is given either alone, or in combination, it exhibits significantly higher success rates as compared to all other classes of drugs (Table 3). This held true when a beta blocker was given in 2 drug (Table 4), or 3 drug combinations (Table 5). It is evident that while our endpoint was successful treatment of hypertension within 90 days, the Cochrane analyses looked at long‐term cardiovascular and other causes of mortality and morbidity. However, as these serious events are directly related to the uncontrolled hypertension, then patients with good adherence should benefit from the inclusion of a beta blocker in their combination arsenal. Our results, stemming from machine learning of a large and diverse set of big data, in contrast to the much narrower criteria for RCTs, should be corroborated and affirmed by other methods, as they hold potential promise for an old class of drugs which may be presently underutilized.

In the second part of our study we wished to identify other drugs taken by the same patients, which may have capacity to decrease BP beyond what is expected from the classical antihypertensive drugs. This analysis reveals that 2 classes of drugs commonly used by patients with hypertension, appear to exert an independent BP lowering effects: the PPI and the HMG CO‐A reductase inhibitors (statins). These effects were robust after careful propensity score matching and correction for multiple comparisons.

In both cases, these potential effects have not been realized till recently by mainstream clinical practice and research. In 2009 Bautista compared BP in statin users and nonusers from the National Health and Nutrition Examination Surveys.^13^ The overall effect of statins and their interactions with antihypertensive medication, BP, and HDL‐C were estimated. Adjusted systolic BP was on average 1.8 mm Hg lower in statins users than in nonusers. Although statins had no significant effect among nonusers, it significantly decreased systolic BP by 3.3 mm Hg among users of antihypertensive medications. The effect of statins on systolic BP was similar in individuals with HDL‐C levels above and below the median. Statins also lowered diastolic BP by an average of 1.9 mm Hg regardless of antihypertensive medication use. Among individuals with high HDL‐C, statins did not lower diastolic BP, whereas in those with low HDL‐C diastolic BP was reduced by 3.4 mm Hg. The effect of statins on systolic and diastolic BP increased with higher BP and changed little with adjustment for total cholesterol. Bautista concluded that the effect of statins on hypertension is through a mechanism different from their lipid lowering effects.^13^

In a recent meta‐analysis, there was a significant reduction in plasma asymetric dimethylarginine (ADMA) by statins. Endothelial dysfunction may be associated with increased circulating ADMA,^14^ and this may be a potential mechanism for this BP lowering effect of statins.

In 2014 Joya‐Vazquez and colleagues analyzed records of hypertensive patients according to regular use of PPI.^15^ While measuring BP continuously by 24 hour ambulatory BP monitoring, both systolic and diastolic BPs were significantly lower among PPI users. In multivariate analysis, the use of beta blockers (OR 2.60) and PPI (OR 2.70) were independently associated with BP control. Mechanistically, PPI have been shown to reduce vascular tone in experimental systems.^16^

In summary, these previously unrecognized effects of PPIs and statins have been very recently identified in preliminary clinical observations, lending credibility to our novel data science methodology. This experience suggests that data science methodology using machine learning may be an effective means for repurposing medications already on the market, for new indications.

## DISCLOSURES

None declared.
